# Prenatal echocardiography diagnosis of a novel combination of bilateral ductus arteriosus and cardiovascular anomalies: a case report and literature review

**DOI:** 10.3389/fcvm.2024.1389759

**Published:** 2024-05-09

**Authors:** Simin Zhang, Ning Wang, Pengfei Qu, Xiaobin Shu, Yang Mi, Xinru Gao

**Affiliations:** ^1^ Ultrasonic Diagnosis Center, Northwest Women’s and Children’s Hospital, Xi’an, Shaanxi, China; ^2^ Translational Medicine Center, Northwest Women’s and Children’s Hospital, Xi’an, Shaanxi, China; ^3^ Department of Obstetrics and Gynecology, Northwest Women’s and Children’s Hospital, Xi’an, Shaanxi, China

**Keywords:** echocardiography, fetus, bilateral ductus arteriosus, right aortic arch, mirror-image branching, dextro-transposition of the great arteries, spatiotemporal image correlation

## Abstract

**Background:**

Bilateral ductus arteriosus (BDA) is a relatively rare vascular malformation. According to the double arch theory, BDA is formed when the distal ends of the sixth pairs of primitive arches on the left and right sides have not regressed. We describe a fetus with prenatal echocardiographic findings of BDA and right aortic arch mirror-image branching (RAA-MIB) combined with congenital heart disease. Furthermore, to gain a deeper understanding of the embryological mechanism of BDA, we review the literature on all combinations of BDA present in 40 fetuses/infants.

**Case summary:**

A 22-year-old female patient underwent fetal echocardiography at 23 weeks of gestation. Both the two-dimensional (2D) grayscale image and color Doppler flow imaging (CDFI) revealed dextro-transposition of the great arteries combined with a ventricular septal defect and RAA-MIB. The following scan revealed a rare vascular ring, which was identified as BDA extending from the confluent of the left pulmonary artery and right pulmonary artery, completely encircling the trachea to form an “O”-shaped vascular ring before finally converging into the descending aorta. A persistent left superior vena cava was also observed. We subsequently used four-dimensional (4D) color Doppler imaging with the spatiotemporal image correlation (STIC) HD live flow and STIC HD live flow silhouette mode to clearly display ventricular arterial connectivity and the direction of vessel travel. Adjusting the image quality and display angle is very important when applying STIC. The 4D images confirmed our diagnosis. After multidisciplinary counseling and discussion with her family, this female patient decided to terminate the pregnancy.

**Conclusion:**

Our review of the literature summarized nine combinations classified into three types of BDA and aortic arch pathology. However, our case differs because it is a novel combination of intracardiac structural abnormalities and vascular rings in a fetus. Prenatal ultrasound diagnosis of BDA is important and requires a combination of 2D grayscale, CDFI, and STIC images to assist in scanning.

## 1 Introduction

Bilateral ductus arteriosus (BDA) is a relatively rare vascular malformation. In most neonatal cases, BDA is not patented and is accompanied by pulmonary atresia (PA) and a non-confluent pulmonary artery. Based on Edward's hypothesis of the double arch theory, BDA forms when the distal ends of the sixth pairs of primitive arches on the left and right sides have not regressed (1).

We describe the case of a fetus with prenatal echocardiographic findings of BDA and right aortic arch mirror-image branching (RAA-MIB), in addition to dextro-transposition of the great arteries (d-TGA) and a persistent left superior vena cava (PLSVC). This rare vascular ring is likely to compress the trachea and esophagus. We report a novel association between intracardiac structural abnormalities and vascular rings in a fetus. Furthermore, we review the literature on all combinations of BDA in fetuses and infants to gain a deeper understanding of the embryological mechanism of BDA and to highlight the novel combination of BDA observed in the present case.

## 2 Case presentation

**Figure 1:**
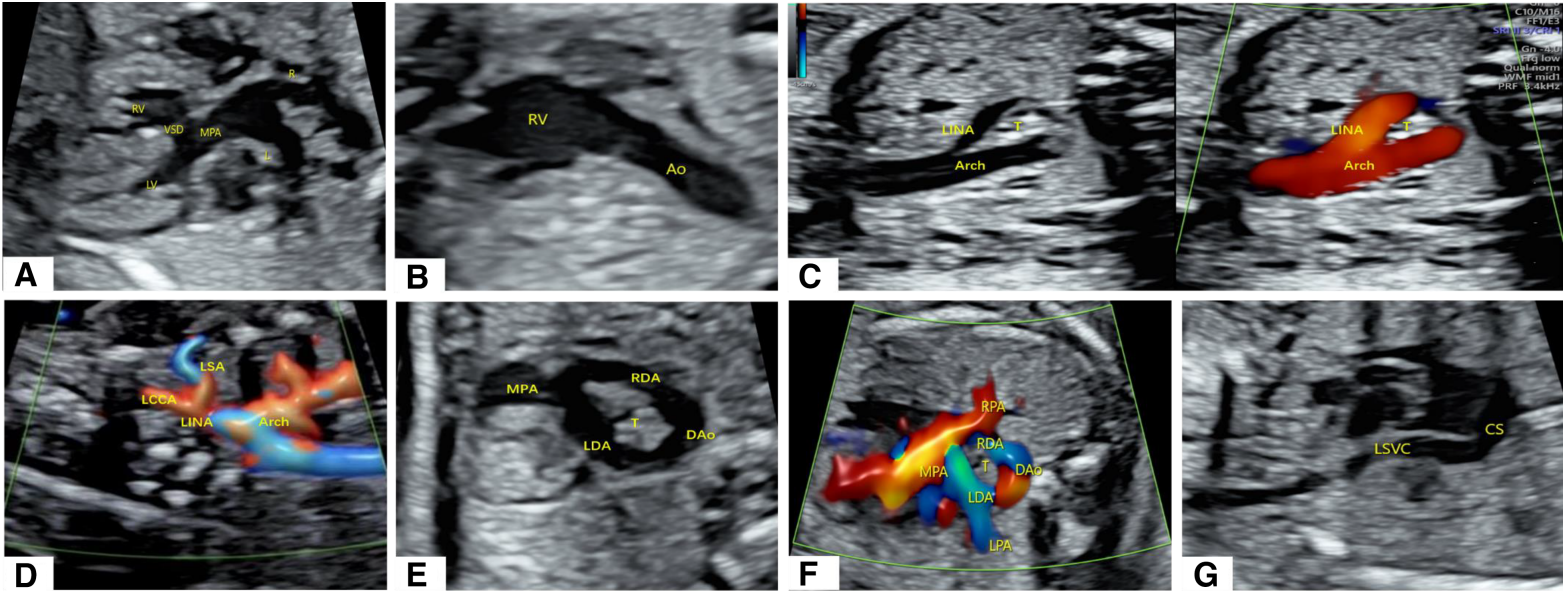
Abnormal cardiac structure and vascular abnormalities in the fetus. (**A**,**B**) 2D grayscale images showing d-TGA combined with a VSD. (**C**,**D**) 2D grayscale combined with CDFI showing RAA-MIB. (**E**,**F**) 2D grayscale combined with CDFI showing BDA encircling the trachea. (**G**) 2D grayscale showing that the PLSVC converges into the CS.

A 22-year-old female patient, Gravida 3, Para 2 (G3P2), underwent fetal echocardiography at 23 weeks of gestation. Both the two-dimensional (2D) grayscale image and color Doppler flow imaging (CDFI) revealed cardiac structural abnormalities. The 2D grayscale imaging showed that the main pulmonary artery (MPA) originated from the left ventricle (LV) and could be seen overriding the interventricular septum, with left and right blood vessels separating at the distal end (Figure 1A). Furthermore, the aorta (Ao) was shown to originate completely from the right ventricle (RV) (Figure 1B). The above signs indicated that the fetus had d-TGA combined with a ventricular septal defect (VSD). 2D grayscale combined with CDFI showed that the Ao was located on the right side of the trachea and that the first blood vessel originating from the Ao was the left innominate artery (LINA) (Figure 1C), which was toward the left shoulder of the fetus. Subsequently, the left common carotid artery (LCCA) and left subclavian artery (LSA) were separated (Figure 1D), indicating RAA-MIB (Supplementary Video S1). The following scan revealed a rare vascular ring, which extended from the confluent of the left pulmonary artery (LPA) and right pulmonary artery (RPA) to the left and right ductus arteriosus (LDA and RDA), converging into the descending aorta (DAo). Therefore, the BDA completely encircled the trachea, forming an “O”-shaped vascular ring (Figures 1E,F, Supplementary Video S1). In addition, a PLSVC was observed; the 2D grayscale longitudinal plane showed that this vessel converged into the coronary sinus (CS) (Figure 1G). We have provided a pattern diagram to help understand this case (Supplementary Figure S1).

**Figure 2:**
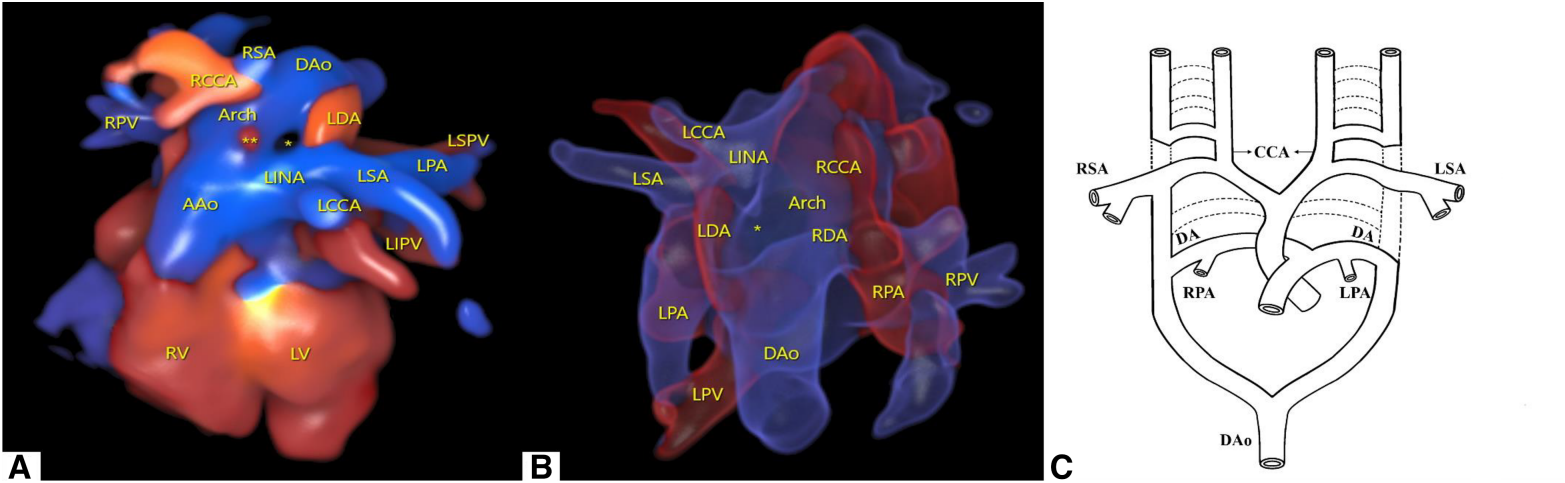
Cardiovascular abnormalities of this fetus observed using the STIC HD live flow mode and embryonic development patterns based on Edward's double aortic arch system theory. (**A**) Shown from the anterior view, we used STIC HD live flow mode to observe that the first blood vessel originating from the ascending aorta was the LINA, which then separates into the LCCA and LSA, followed by the RCCA and RSA (*indicates the “O”-shaped vascular ring, ** indicates red blood flow indicates the RDA). (**B**) Shown from the posterior view, we used STIC HD live flow Silhouette mode to find the RDA (*indicates the “O”-shaped vascular ring). (**C**) Embryonic development patterns based on Edward's double aortic arch system theory.

To better visualize the spatial effect, we used a four-dimensional (4D) color Doppler with spatiotemporal image correlation (STIC) HD live flow mode (Voluson E10, GE Healthcare, Zipf, Austria) to clearly display ventricular arterial connectivity and the direction of vessel travel. From this three-dimensional (3D) image, it can be inferred that in the d-TGA, the RAA with LDA formed a “U”-shaped vascular ring. At this point, the RDA was located immediately below the RAA and could only be observed with flickering of the RDA in certain cardiac cycles (Figure 2A, Supplementary Video S2).

Furthermore, we utilized the STIC HD live flow Silhouette mode (CFM Silhou.90, CFM thresh.30, Transp. CFM 20) to identify the RDA hidden beneath the RAA via a posterior view, which required adjustments in the image quality and angle when applying STIC. The LDA and RDA originated from the proximal end of the LPA and RPA, respectively, and converged into the DAo. Different blood flow directions could help distinguish between pulmonary arteries and veins. Due to angle factors, only the first branch of the Ao could be seen (Figure 2B, Supplementary Video S3). Following multidisciplinary counseling and discussions with her family, this female patient decided to terminate her pregnancy and refused to undergo genetic testing.

## 3 Discussion

To the best of our knowledge, this is the first report of BDA combined with RAA-MIB. Furthermore, there are no reports of the above combined with d-TGA, as shown in the pattern diagram (Supplementary Figure S1). As known, an “O”-shaped vascular ring often occurs in a double aortic arch. Usually, the right side arch is larger than the left side arch; however, the “O”-shaped vascular ring in our case showed that the diameters of the DA on both sides were almost identical. This was a good identification point on ultrasound images, and the spatial position of the DA was lower than that of the aortic arch. In more than 98% of cases, the RAA-MIB is closely related to cardiac abnormalities, including tetralogy of Fallot, persistent truncus arteriosus, and d-TGA (1, 2). Our case is consistent with the facts reported in the literature.

**Figure 3:**
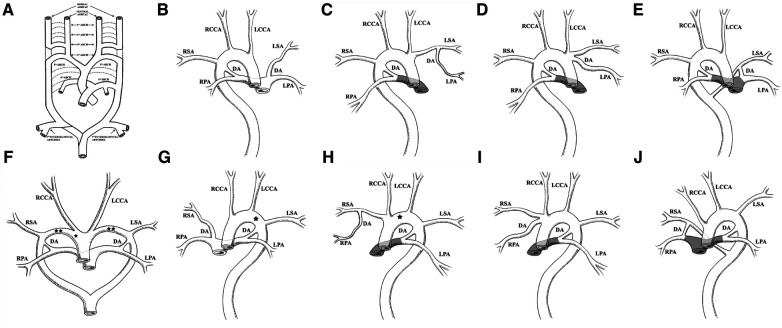
Edward's DAA system theory and the nine combinations of BDA and aortic arch abnormalities summarized in the literature. (**A**) Edward's DAA system theory. The dotted line represents regression, and the black solid line represents the preserved primitive arch artery. Reprinted with permission of Radiological Society of North America, from Hanneman et al. (1). (**B**) RAA combined with an ILSA. The LSA originates from the LPA via the LDA. (**C**) RAA-MIB combined with PA. The LPA originates from the LSA via the LDA. (**D**) RAA-MIB with or without PA. The LPA originates from the LINA via the LDA. (**E**) RAA combined with an aberrant left subclavian artery (ALSA). The LDA is connected to the LPA and ALSA. (**B**–**E**) RDA is connected to the RPA and the DAo. (**F**) DAA combined with BDA. The LDA is connected to the LPA and left arch, and the RDA is connected to the RPA and right arch. *represents interruption of the aortic arch (IAA) occurring in the right arch; **represents IAA occurring on both sides of the aortic arch. (**G**) LAA combined with IRSA. The RSA originates from the RPA via the RDA. *represents IAA occurring in the left arch. (**H**) LAA, RPA originates from the RSA via the RDA, with or without PA. *represents IAA occurring in the left arch. (**I**) LAA, RPA originates from the INA via the RDA, with or without PA. (**J**) LAA combined with ARSA, with or without PA. The RDA is connected to RPA and ARSA, respectively. (**G**–**J**) LDA is connected to the LPA and the DAo. The shadowed area in panels (**C**–**E**) and **(H**–**J**) represents PA.

The embryological mechanism of development of the aortic arch has always been supported by Edward's theory, namely, the double aortic arch system (3). The value of this theory lies in its ability to explain the potential contribution of almost all embryonic arches to the final adult arch system components (1). As shown in Figure 3A, this is a complex process of development and regression. The primitive aorta is composed of ventral and dorsal parts. Six pairs of primitive arch arteries develop between the ventral and dorsal aortae. The dorsal aortae also give rise to the seventh intersegmental arteries. The appearance and degeneration of the primitive arch artery do not occur simultaneously. Ultimately, the first, second, and fifth pairs of primitive arch arteries degenerate. In normal development, the right proximal sixth arch continues to exist as the proximal part of the RPA, while its distal part degenerates as the DA, in addition to partial degeneration of the right dorsal aorta (dotted line in Figure 3A). The primitive third, fourth, and sixth pairs of arch arteries contribute to the common carotid artery, future aortic arch, proximal pulmonary artery, and DA, respectively. The seventh intersegmental arteries contribute to the subclavian artery (black solid line in Figure 3A) (1, 4, 5).

In the present case, both primitive sixth arches had not degenerated. The partial degeneration of the left dorsal aorta occurs near the sixth and fourth arch arteries; that is, degeneration of the left aortic arch occurs between the LSA and LDA. Therefore, it causes the RDA and LDA to be directly connected to DAo (Figure 2C). The degeneration of different combinations of the primitive fourth and sixth arch arteries can lead to different types of aortic arch and DA.

Based on the findings of this case, we reviewed all literature works on BDA and summarized 40 cases of BDA in 25 articles over the last few decades. Some of the cases in the literature did not provide basic information and imaging findings of the anatomical features of specific cases; therefore, they were not included in the summary in Table 1 (31). The characteristics of these cases are that at least one side of the DA is not directly connected to the DAo, and they are all reported during the fetal or infant periods. Figure 3 can be used to understand the combination forms of these anomalies.

**Table 1:** Basic information and imaging findings of 41 cases.

Case	References	Diagnosis time		Aortic arch position	Type	LDA connection	RDA connection	Associated CHD	EA	Outcomes	Chromosomal test	Bilateral DA close		Figure
		GA (weeks)	Newborn age (days/weeks/months)									Left	Right	
1	Wen et al. (6)	28 weeks		RAA	Ia	LPA-LDA-ILSA	RPA-RDA-DAo	No	No	Born	NM	Yes	Yes	3B
2	Sun et al. (7)	25 weeks		RAA	Ia	LPA-LDA-ILSA	RPA-RDA-DAo	No	No	Born	NM	Yes	Yes	3B
3	Patel et al. (8)	25 weeks		RAA	Ia	LPA-LDA-ILSA	RPA-RDA-DAo	No	No	Born	(–)	NM	NM	3B
4	Freedom et al. (9)		Newborn	RAA	Ia	LPA-LDA-ILSA	RPA-RDA-DAo	NM	NM	Death	NM	NM	NM	3B
5	Abdul Latiff et al. (10)		25 days	RAA-MIB	Ib1	LPA-LDA-LSA	RPA-RDA-DAo	PA, VSD	No	Survival	NM	No	No	3C ^a^
6	Karmegaraj and Vaidyanathan (11)	19 weeks		RAA-MIB	Ib2	LINA-LDA-LPA	RPA-RDA-DAo	No	No	TOP	NM	NM	NM	3D
7	van Velzen et al. (12)	22 weeks		RAA-MIB	Ib2	LINA-LDA-LPA	RPA-RDA-DAo	No	No	Born	(–)	Yes^b^	Yes^b^	3D
8	Freedom et al. (9)		Newborn	RAA-MIB	Ib2	LINA-LDA-LPA	RPA-RDA-DAo	No	No	NM	NM	NM	NM	3D
9	Kearney et al. (13)		1 day	RAA-MIB	Ib2	LINA-LDA-LPA	RPA-RDA-DAo	AVSD, PTA, CTA	HTX	Death	NM	No^c^	No^c^	3D ^a^
10	Lenox et al. (14)		1 day	RAA-MIB	Ib2	LINA-LDA-LPA	RPA-RDA-DAo	d-TGA, SV, PA, MA	No	Survival	NM	No	No	3D ^a^
11	Murray et al. (15)		9 months	RAA-MIB	Ib2	LINA-LDA-LPA	RPA-RDA-DAo	PTA, VSD	No	Death	NM	No	No	3D ^a^
12	Park et al. (16)		34 days	RAA-MIB	Ib2	LINA-LDA-LPA	RPA-RDA-DAo	Dextrocardia, PA, AVSD	HTX	Survival	NM	No	No	3D ^a^
13			11 days	RAA-MIB	Ib2	LINA-LDA-LPA	RPA-RDA-DAo	cc-TGA, PA	SI	Survival	NM	No	No	3D ^a^
14	Freedom et al. (9)		Newborn	RAA	Ic	LPA-LDA-ALSA	RPA-RDA-DAo	PA	NM	Death	NM	NM	NM	3E ^a^
15	Ma et al. (17)	25 weeks		RAA	Ic	LPA-LDA-ALSA	RPA-RDA-DAo	No	No	Born	(–)	Yes	Yes	3E
16	Lenox et al. (14)		6 days	RAA	Ic	LPA-LDA-ALSA	RPA-RDA-DAo	d-TGA/IVS	No	Survival	NM	No	No	3E
17	Lenox et al. (18)		2 days	RAA	Ic	LPA-LDA-ALSA	RPA-RDA-DAo	d-TGA/IVS	No	Survival	NM	No	No	3E
18	Yuksel et al. (19)	16 weeks		RAA	Ic	LPA-LDA-ALSA	RPA-RDA-DAo	AVSD	ACC,PUV	TOP	T18	NM	NM	3E
19	Shirali GS et al. (20)		1 day	DAA	III	LPA-LDA-LAA	RPA-RDA-RAA	d-TGA/IVS	No	Survival	NM	No	No	3F
20	Dipchand et al. (21)		Newborn	DAA	III	LPA-LDA-LAA	RPA-RDA-RAA	IAA(RAA), AVSD,	HTX	Survival	NM	No	No	3F ^d^
21	Blatchford et al. (22)		3 days	DAA	III	LPA-LDA-LAA	RPA-RDA-RAA	IAA(DAA), VSD	No	Death	NM	No	No	3F ^e^
22	Keagy et al. (23)		16 months	LAA	IIa	LPA-LDA-DAo	RPA-RDA-IRSA	No	No	Death	NM	No	No	3G
23			7 days	LAA	IIa	LPA-LDA-DAo	RPA-RDA-IRSA	IAA, VSD	No	Survival	NM	NM	NM	3G
24	Nath et al. (24)		Newborn	LAA	IIa	LPA-LDA-DAo	RPA-RDA-IRSA	DORV, VSD	No	Survival	NM	NM	NM	3G
25			18 months	LAA	IIa	LPA-LDA-DAo	RPA-RDA-IRSA	No	No	Survival	NM	NM	NM	3G
26			Newborn	LAA	IIa	LPA-LDA-DAo	RPA-RDA-IRSA	d-TGA, VSD	No	Survival	NM	NM	NM	3G
27	Kumar et al. (25)		4 months	LAA	IIa	LPA-LDA-DAo	RPA-RDA-IRSA	IAA (B type), VSD	No	Survival	NM	No	No	3G ^d^
28	Barger et al. (26)		7 days	LAA	IIa	LPA-LDA-DAo	RPA-RDA-IRSA	IAA (B type), VSD	No	Death	NM	NM	NM	3G ^d^
29	Jew and Gross (27)		Newborn	LAA	IIb1	LPA-LDA-DAo	RPA-RDA-RSA	No	No	NM	NM	NM	NM	3H
30	Freedom et al. (9)		Newborn	LAA	IIb1	LPA-LDA-DAo	RPA-RDA-RSA	PA, SV, MA	No	Survival	NM	No	No	3H ^a^
31			Newborn	LAA	IIb1	LPA-LDA-DAo	RPA-RDA-RSA	IAA (type C)	No	NM	NM	NM	NM	3H ^d^
32	Han et al. (28)	22 weeks		LAA	IIb2	LPA-LDA-DAo	INA-RDA-RPA	No	No	TOP	(–)	NM	NM	3I
33	Lenox et al. (14)		5 days	LAA	IIb2	LPA-LDA-DAo	INA-RDA-RPA	SA, SV, PA	No	Death	NM	No	No	3I
34			1 day	LAA	IIb2	LPA-LDA-DAo	INA-RDA-RPA	VSD, PLSVC	NM	NM	NM	NM	NM	3I
35	Chen Let al. (29)	25 weeks		LAA	IIb2	LPA-LDA-DAo	INA-RDA-RPA	No	NM	NM	NM	NM	NM	3I
36	Murray et al. (15)		3 weeks	LAA	IIb2	LPA-LDA-DAo	INA-RDA-RPA	PTA, VSD	No	Death	NM	No	No^f^	3I
37	Abdul Latiff yet al. (10)		15 days	LAA	IIb2	LPA-LDA-DAo	INA-RDA-RPA	Dextrocardia, TA, VSD, PA	No	Survival	NM	No	No	3I ^a^
38	Freedom et al. (9)		Newborn	LAA	IIb2	LPA-LDA-DAo	RPA-RDA-RSA	No	NM	NM	NM	NM	NM	3I
39			Newborn	LAA	IIc	LPA-LDA-DAo	RPA-RDA-ARSA	PA, VSD	No	NM	NM	NM	NM	3J ^a^
40	Wang et al. (30)	23 weeks		NM	NM	LPA-LDA-LSA	RPA-RDA-DAo	SV,SA,DORV, IAA, DSVC	No	NM	NM	NM	NM	NM
Our case														
41	Zhang et al.	23 weeks		RAA-MIB	III	LPA-LDA-DAo	RPA-RDA-DAo	d-TGA, VSD, PLSVC	No	TOP	No conducted	No	No	2C

GA, gestational age; DA, ductus arteriosus; CHD, congenital heart disease; EA, extracardiac abnormalities; RAA, right aortic arch; LPA, left pulmonary artery; ILSA, isolated left subclavian artery; RPA, right pulmonary artery; DAo, descending aorta; NM, not mentioned; RAA-MIB, right aortic arch mirror-image branching; PA, pulmonary atresia; VSD, ventricular septal defect; TGA, transposition of the great arteries; IVS, interventricular septum; d-, dextro-; c-, complete; cc-, corrected; ALSA, aberrant left subclavian artery; ACC, abnormal corpus callosum; PUV, posterior urethral valves; TOP, termination of pregnancy; T18, trisomy 18; LINA, left innominate artery; AVSD, atrioventricular septal defect; PTA, persistent truncus arteriosus; CTA, cor triatriatum; HTX, heterotaxy syndrome; SV, single ventricle; MA, mitral atresia; SI, situs inversus; DAA, double aortic arch; IAA, interruption of aortic arch; IRSA, isolated right subclavian artery; DORV, double outlet right ventricle; LAA, left aortic arch; RSA, right subclavian artery; ARSA, aberrant right subclavian artery; SA single atrium; PLSVC, persistent left superior vena cava; TA, tricuspid atresia; DSVC, double inferior vena cava.

^a^ Representing main pulmonary artery atresia, absence or PTA.

^b^ Prostaglandin E1 infusion was started to prevent DA closure. In 3H, both left and right pulmonary arteries are connected to the main pulmonary artery, while the RPA is connected to RSA via RDA.

^c^ Pathology confirmed that both left and right pulmonary arteries were arterial ductal tissue, and there was an absence of both pulmonary arteries.

^d^ Representing IAA.

^e^ Representing IAA on both side arches.

^f^ Pathological confirmation of arterial ductal tissue.

We have summarized the nine combinations of BDA and aortic arch, which mainly include the right aortic arch (RAA), double aortic arch (DAA), and left aortic arch (LAA), and classified the combinations into three types. Type I refers to the RDA of the RAA connected to the RPA and DAo, with the LDA being ectopic. Type II refers to the LDA of the LAA connected to the LPA and DAo, with the RDA being ectopic. Type III refers to the bilateral DA connected to the aortic arch or near the DAo, with the other end connected to the pulmonary artery. Under each type, subtypes were classified as follows: a is the aortic branch, which is not connected to the aortic circulatory system originating from the pulmonary circulatory system via the DA; b refers to the pulmonary artery branches originating from the aortic circulatory system via the DA; and c usually occurs when the pulmonary artery is connected to the aberrant subclavian artery via the DA.

We refer to Type Ia, named isolated left subclavian artery (ILSA), as LPA-LDA-ILSA (Figure 3B) (6–9). Type Ib1 is characterized when the RAA-MIB is combined with PA, and the LPA originates directly from the LSA via the LDA; we refer to it as LPA-LDA-LSA (Figure 3C) (10). Type Ib2 occurs when RAA-MIB is present with or without PA, and the LPA originates from the LINA via the LDA; we refer to it as LPA-LDA-LINA (Figure 3D) (13–16). Type Ic occurs when the LDA is connected to the LPA and the aberrant LSA (ALSA); we refer to it as LPA-LDA-ALSA (Figure 3E) (10, 14, 17–19). In type I, the RDA mentioned above are all connected to the RPA and Dao; we refer to it as RPA-RDA-DAo.

We refer to Type IIa, named isolated right subclavian artery (IRSA), as RPA-RDA-IRSA (Figure 3G) (23–26). In Type IIb1, the RPA originates directly from the RSA via the RDA, with or without PA; we refer to it as RPA-RDA- RSA (Figure 3H) (10, 27). In Type IIb2, the RPA originates from the INA via the RDA, with or without PA; we refer to it as RPA-RDA-INA (Figure 3I) (9, 10, 14, 15, 28–30). In Type IIc, the RDA is connected to the RPA and aberrant RSA (ARSA), with or without PA; we refer to it as RPA-RDA-ARSA (Figure 3J) (10). In Type II, the LDA mentioned above are all connected to the LPA and Dao; we refer to it as LPA-LDA-DAo.

In Type III, we summarized two situations. The first one is our case, which involves an RAA with a bilateral DA connected to the Dao; we refer to it as LPA-LDA-DAo and RPA-RDA-DAo. Another type is DAA, where the LDA is connected to the LPA and LAA and the RDA is connected to the RPA and RAA; we refer to it as LPA-LDA-LAA and RPA-RDA-RAA (Figure 3F). The combination of DAA and BDA is very rare (20). In addition, two cases were found with this combination, one with interruption of the aortic arch (IAA) on the right side (21) and the other with a double side (22).

Coincidentally, Ia and IIa, Ib and IIb, and Ic and IIc almost form a mirror effect (Figures 3B and G, C and H, D and I, and E and J), which helps us to better understand and remember these patterns. In most cases, ectopia is always on one side of the BDA, almost occurring on the opposite side of the aortic arch, which seems to have some potential association. If there is PA, the blood supply to the pulmonary artery branch by the aorta is via the DA. At this point, the DA is defined as an abnormal connection rather than an ectopic connection. In our case, it is rare for the BDA to directly connect to the DAo without an ectopic connection, as we emphasized earlier.

In Table 1, 41 cases of BDA were included (including the cases in our study). Only 11 cases were reported during the fetal period (26.8%, 11/41). The age range of the other reported cases is from 1 day to 18 months after birth. Among the 41 cases, 26 (63.4%, 26/41) had congenital heart disease (CHD), 13 (31.7%, 26/41) did not have CHD, and the presence of CHD was not mentioned in the others. Among the 26 cases of CHD, there were 9 cases of PA, 7 cases of IAA, 6 cases of TGA, 4 cases of AVSD, 3 cases of PTA, and 3 cases of SV. These cases of PA were all reported in the infant period. In addition, in cases where birth outcomes could be followed up, DA closure occurred in 4 cases of RAA without CHD; however, in the other 17 cases, the DA was not closed, and all cases were combined with CHD. Among them were seven cases of PA, indicating that the DA provides the blood supply to both lungs. Because many studies have only reported the cardiac manifestations, the summary of extracardiac anomalies is not comprehensive. Heterotaxy syndrome (HTX) has the highest incidence, with three cases reported, which is similar to previous studies findings (9, 31). HTX is a laterality defect. In recent years, many scholars believe that TGA and AVSD are also laterality defects. In addition, most HTX patients also have PA (32). Our summary in Table 1 found that the highest incidence of CHD in BDA is PA, TGA, AVSD, etc. In addition, BDA always appears heterotopic on the opposite side of the aortic arch. The above clues indicate that TGA with BDA could further support the hypothesis of TGA as a form of latency defect.

In summary, it is necessary to pay attention to BDA. Our case demonstrates a novel combination of BDA and cardiovascular anomalies. In addition, we focused on the forms of BDA reported in the current literature. During prenatal echocardiography, special attention should be paid to the position of the aortic arch and vascular branches, and the presence of ectopic DA should be observed on the opposite side of the aortic arch. Furthermore, the cardiac structure should be observed when complex vascular rings appear. The use of STIC can effectively display the 3D structures; however, it is still necessary to combine 2D grayscale images and CDFI to demonstrate spatial relationships well.

## Data Availability

The original contributions presented in the study are included in the article/**Supplementary Material**, further inquiries can be directed to the corresponding authors.

## Supplementary material

Supplementary Video S1 Color Doppler flow imaging of BDA with RAA-MIB. The BDA completely encircles the trachea formed an “O”-shaped vascular ring. Above it is the Ao, and LINA can be seen.

Supplementary Video S2 4D color Doppler with STIC HD live flow mode was showed in this case. It can be inferred that d-TGA and the RAA with LDA formed a “U”-shaped vascular ring. At this point, the RDA was located immediately below the RAA and can only be observed with flickering RDA in certain cardiac cycles.

Supplementary Video S3 STIC HD live flow Silhouette mode was showed in this case. The posterior view shows that we used the STIC HD live flow Silhouette mode to find the RDA hidden beneath the RDA.

Supplementary Figure S1 The cardiovascular abnormalities of this fetus in pattern diagram are presented. The abnormal vascular features and abnormal intracardiac structure of this case are shown.
