# Supplementary figures and images for: Prenatal echocardiography diagnosis of a novel combination of bilateral ductus arteriosus and cardiovascular anomalies: a case report and literature review
Source: Front Cardiovasc Med. 2024 May 9;11:1389759. doi: 10.3389/fcvm.2024.1389759 (PMC11111948; doi:10.3389/fcvm.2024.1389759)

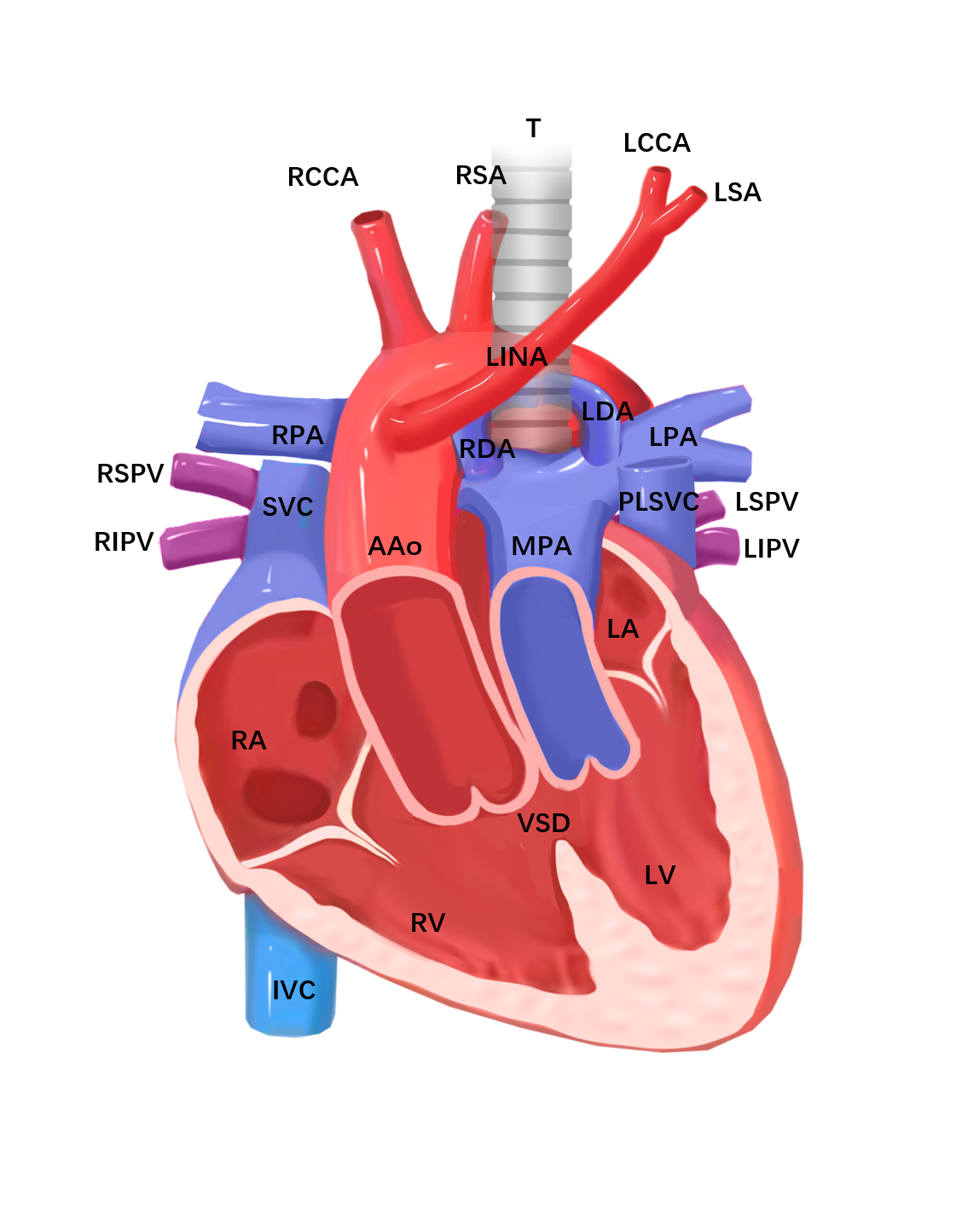

Supplement: Supplementary Figure S1 — The cardiovascular abnormalities of this fetus in pattern diagram are presented. The abnormal vascular features and abnormal intracardiac structure of this case are shown. [file Image1.tif]
